# Comparison of Laparoscopic Intraperitoneal Onlay Mesh Repair (IPOM plus) vs Open Rives–Stoppa (RS) Repair for Ventral and Incisional Hernia

**DOI:** 10.15388/Amed.2024.31.2.11

**Published:** 2024-12-04

**Authors:** Hryhorii O. Havrylov, Oleg V. Shulyarenko, Mykhaylo O. Yosypenko

**Affiliations:** 1 Clinic “Medikom”, Kyiv, Ukraine; 2 Department of General Surgery #2, Bogomolets National Medical University, Kyiv, Ukraine; 3 Department of Surgery and Proctology, Shupyk National Healthcare University of Ukraine, Kyiv, Ukraine

**Keywords:** laparoscopy, hernia, mesh, surgery, IPOM plus, Raktažodžiai: laparoskopija, išvarža, tinklelis, operacija, IPOM plius

## Abstract

**Aim:**

To compare the effects of laparoscopic intraperitoneal onlay mesh plus repair versus open Rives–Stoppa repair for abdominal wall hernias.

**Materials and methods.:**

A total of 99 patients with midline primary, umbilical or incisional hernias who underwent surgery in “Medikom” clinic and Kyiv city hospital #5 in the period from 2016 to 2022 were involved in the study. The group I included 50 patients who underwent intraperitoneal onlay mesh (IPOM) plus, and the group II 49 patients who underwent open Rives–Stoppa (RS) repair.

**Result:**

Both groups were comparable in mean age, gender, body mass index, patient distribution based on hernia type, defect size, ASA score distribution (p > 0.05).

The operating time in minutes was 75.36 ± 4.99 in group I and 97.85 ± 6.5 in group II (p < 0.05). The blood loss in IPOM plus approach group is on average in 2 times less than in open RS technique (p < 0.05). The pain score 12 hours after surgery was 5.5 ± 0.64 in group I comparing to 7.26 ± 0.78 in group II (p < 0.05). The pain score 24 hours after surgery was 4.46 ± 0.7 in group I comparing to 4.95 ± 0.61 in group II (p < 0.05). The pain score 8 days after surgery in group I was on average in 1.46 times less than in group II (p < 0.05).

No significant difference was found in incidence of early complications between two groups (p > 0.05).

47 (94%) patients of group I and 46 (93.87%) patients of group II were evaluated over 22 months follow-up period. No one complication was noted.

**Conclusions.:**

IPOM plus approach for the ventral and incisional hernias repair is a viable and relatively more safe operation by comparing with open RS repair. IPOM plus approach takes significantly in 1.3 less time as compared open RS technique.

## Abstract (it)

**Tikslas:**

Palyginti laparoskopinio intraperitoninio tinklelio korekcijos ir atviros Rives-Stoppa korekcijos poveikį pilvo sienos išvaržų atveju.

**Metodai:**

Iš viso tyrime dalyvavo 99 pacientai, kuriems 2016–2022 m. „Medikom“ klinikoje ir Kiyvo miesto ligoninėje Nr. 5 buvo operuotos vidurinės linijos pirminės, bambos ar incizinės išvaržos. į I grupę pateko 50 pacientų, kuriems buvo atliktas intraperitoninio tinklelio tvirtinimas (IPOM plus), o į II grupę – 49 pacien-tai, kuriems buvo atliktas atviras Rives-Stoppa (RS).

**Rezultatai:**

Abiejų grupių pacientų amžiaus, lyties, kūno masės indekso, pacientų pasiskirstymo pagal išvaržos tipą, defekto dydį, ASA balų pasiskirstymą rodikliai buvo panašūs (p > 0,05).

Operacijos trukmė minutėmis I grupės pacientų buvo 75,36±4,99, o II grupės – 97,85±6,5 (p < 0,05). Kraujavimas IPOM plius metodo grupėje vidutiniškai du kartus mažesnis nei taikant atvirą RS metodą (p < 0,05). Skausmo balas praėjus 12 valandų po operacijos I grupėje buvo 5,5±0,64, palyginti su 7,26±0,78 II grupėje (p < 0,05). Skausmo balas praėjus 24 valandoms po operacijos I grupėje buvo 4,46±0,7, palyginti su 4,95±0,61 II grupėje (p < 0,05). Skausmo balas praėjus 8 dienoms po operacijos I grupėje buvo vidutiniškai 1,46 karto mažesnis nei II grupėje (p < 0,05).

Ankstyvųjų komplikacijų dažnio reikšmingo skirtumo tarp dviejų grupių nenustatyta (p > 0,05).

47 (94 %) I grupės pacientų ir 46 (93,87 %) II grupės pacientų būklė įvertinta per 22 mėnesių stebėjimo laikotarpį. Nebuvo nė vienos komplikacijos.

**Išvados:**

IPOM plius metodas ventralinėms ir incizinėms išvaržoms šalinti yra perspektyvi ir santykinai saugesnė operacija, palyginti su atviru RS šalinimu. IPOM plius metodas užtrunka 1,3 karto trumpiau, palyginti su atviru RS metodu.

## Introduction

The formation of hernias is based on collagenopathy and violations of the abdominal wall biomechanics [1,2]. Each year, more than 20 million hernia repairs are performed around the world [3]. Treatments, also surgery of hernias have also evolved over thousands of years [4]. Management pattern for ventral and incisional hernias are heterogeneous, often with little supporting evidence or correlation with existing evidence [5,6].

Laparoscopy approach was first time described for hernia treatment and proved safe and highly effective in 1992 [7,8].

## The aim

To compare the effects of intraperitoneal onlay mesh plus repair versus open Rives–Stoppa repair for abdominal wall hernias.

## Materials and methods

A total of 99 patients with midline primary, umbilical or incisional hernias with a defect of 2–10 cm who underwent surgery in “Medikom” clinic and Kyiv city hospital #5 in the period from 2016 to 2022 were included in this study. The group I included 50 patients who underwent intraperitoneal onlay mesh (IPOM) plus repair, and group II 49 patients who underwent open Rives–Stoppa (RS) repair. Patients’ age, body mass index (BMI), American society of anesthesiologists (ASA) score patients distribution, gender, hernia type (umbilical hernia, linea alba hernia, incisional hernia) were collected. The patients with ASA IV score and more were excluded in this study. Preoperatively each patient was evaluated performing physical, instrumental examination, also using abdominal ultrasonography (to measure the size of the defect). All procedures were performed with general anesthesia. We evaluated time spent on surgery, blood loss during surgery, postoperative pain level, complications. In the current study we used visual analogue scale (VAS) to define the pain level from 0 to 10 points (painless to severe pain). The pain scores were assessed on the 12 hours, 24 hours, and 8th day after operation. The IPOM plus procedure was performed according to Patent of Ukraine #119299 with Parietene Composite mesh, it involved placement of a mesh with an overlap of 3–5 cm at the edges of the defect, the mesh fixation was performed by absorbable tacks after additional full-sickness sutures using nonabsorbable monofilament [9]. We used polypropylene mesh in open RS procedure, the mesh fixation was performed by polypropylene sutures in separated between rectus abdominis muscle and its posterior vagina space [10,11]. Due to the extensive mobilization done in operation, the vacuum drain was placed in each patient of group II.

After discharge, the patients were called for follow-up at 8 days, 1 month and 3 months thereafter. Patients were reminded about their follow-up appointments by phone calls. The follow-up of the patients was ranged from 4 months to 22 months.

The independent t-test was used to compare age, defect size, body mass index, blood loss, operating time, severity of postoperative pain after 12 hours, 24 hours, 8 days after surgery. The statistics included patient distribution on hernia type, gender, postoperative complications, ASA score distribution which were analyzed by chi-square test (χ2). p<0,05 was considered statistically significant. It also included mean, standard deviation (SD), median; p<0.05 was considered statistically significant.

## Results

**Table 1:** Patient demographics and patient distribution in both groups

Variables	Group I (n = 50)	Group II (n = 49)	p-value
Age, years	48.78 ± 6.42	50.16 ± 6.69	0.29
Body mass index, kg/m2	26.41 ± 1.25	27.01 ± 1.29	0.08
Male : female	28:22	26:23	0.77
Umbilical : linea alba : incisional hernia	11:26:13	9:27:13	0.9
Defect size (width in cm)	5.36 ± 1.48	5.01 ± 1.17	0.19
ASA score distribution I:II:III	8:30:12	9:29:11	0.94

Table 1 shows that the differences in mean age, body mass index, gender, patient distribution based on hernia type, ASA score, defect size were not statistically significant between the two groups (p>0.05). So, both groups were comparable.

Linea alba hernia seems to be the most common hernia in both groups.

**Table 2:** Perioperative details

Variables	Group I (n = 50)	Group II (n = 49)	p-value
Operating time (min)	75.36 ± 4.99	97.8 5 ±6.5	0.00
Blood loss, ml	19.02 ± 2.09	38.26 ± 6.5	0.00
VAS score 12 h after surgery	5.5 ± 0.64	7.26 ± 0.78	0.00
VAS score 24 h after surgery	4.46 ± 0.7	4.95 ± 0.61	0.00
VAS score 8 day after surgery	0.9 ± 0.46	1.32 ± 0.51	0.00
Short-term postoperative complications	Developed port site seroma – 2	Developed surgical wound hematoma – 2 Developed surgical wound infiltrate – 1	0.41

The operating time in minutes was 75.36 ± 4.99 in group I and 97.85 ± 6.5 in group II (p < 0.05). The difference is statistically significant. So, the IPOM plus approach took less time on average in 1.3 times as compared open RS technique.

The blood loss in IPOM plus approach group is on average in 2 times less than in open RS technique (p < 0.05). The difference is statistically significant.

The pain score 12 hours after surgery was 5.5 ± 0.64 balls in group I comparing to 7.26 ± 0.78 in group II (p < 0.05). The difference is statistically significant.

The pain score 24 hours after surgery was 4.46 ± 0.7 balls in group I comparing to 4.95 ± 0.61 (p < 0.05) in group II. The difference is statistically significant.

The pain score 8 day after surgery in group I is on average in 1.46 times less than in group II (p<0.05). It demonstrates the difference is statistically significant.

Thus, after the first 12, 24 hours and after 8 days, patients in the laparoscopic group experienced significantly less pain than patients in the open surgery group.

Conversion to open surgery was not required among the 50 patients of group I.

No significant difference was noted in early complications incidence in both groups (p > 0.05).

The port-site seroma developed in 2 (4%) cases in group I patients. It was successfully treated using puncture under sonography control.

In group II, the postoperative complications occurred in 3 (6.12%) cases. One of them – developed surgical wound hematoma in 2 patients (incisional hernia showed 2 cases of hematoma formation). It was successfully cured using puncture under sonography control and coagulated. Another one developed surgical wound infiltrate was successfully cured conservatively.

47 (94%) patients of group I and 46 (93.87%) patients of group II were evaluated over 22 months follow-up period. No one complication was noted.

## Discussion

The intraperitoneal placement of polypropylene meshes was discouraged because of possible complications, it was strongly recommended to avoid the direct contact between mesh and bowel [12,13]. The trend of laparoscopic repair for ventral wall hernia led to the popularity of composite or coated mesh in which the polyester or polypropylene is separated from the peritoneal contents by a layer of resorbable biodegradable or a coating material. That’s why we used composite mesh for group I patients: on one side a macroporous mesh to repair ventral hernia defects, on the other, an absorbable synthetic film to minimize unwanted tissue attachment. The polypropylene mesh which is used in the implant is designed to create scar tissue on the abdominal wall that had been weak enough to permit the hernia development. This scar tissue grows into the mesh’s pores and highly effective pulling the mesh in to join the muscle. Additional scar tissue, as well as the mesh is supposed to strengthen the muscle enough to prevent a hernia recurring.

The incidence of seroma formation after laparoscopic ventral abdominal wall hernia repairs varies from 5% to 30% [14]. In our study in group I, the port-site seroma developed in 2 (4%) cases.

The chronic pain can associate with laparoscopic IPOM plus approach. It is defined as a pain that lasts more than 3 months. Some authors reported it in 2–9.5% of laparoscopic IPOM plus repair cases and related to nonabsorbable fixation systems [15]. Chronic pain has not been observed in our series. That’s the reason why we prefer only absorbable fixation devices.

Mesh bulging or “pseudo-recurrence” is a well-known phenomenon after laparoscopic ventral hernia defect repair with the aponeurotic edges diverging on straining manifesting as bulging out of the mesh, it is a significant factor in patients’ dissatisfaction with outcomes [16]. That’s why we suture the defects in the dorsal layer consisting of posterior rectus sheath plus peritoneum as well as the ventral layer. During long-term follow-up, major complications associated with laparoscopic IPOM plus technique or open RS technique have not been reported in our study.

The authors concluded that if the mesh is placed inside the peritoneal cavity, there is a risk of adhesiolysis-related complications and an increased difficulty in repeated surgical interventions [17]. Many meshes with anti-adhesive coatings and special fixing materials are available now, so we used high quality composite mesh, also absorbable tacks is our study as the best choice to prevent possible complications.

We are in agreement with the authors of [18] that traditional IPOM plus surgery requires fixation of the mesh to the ventral abdominal wall which is performed by placing two transfascial sutures at the corners or in the midline of the mesh and using either absorbable tackers in between. After mesh fixation with absorbable tacks we remove two transfascial sutures.

In accordance with these findings, the cost of surgery is higher for laparoscopic procedure, but a shorter hospital stay may make laparoscopic surgery more cost effective [19]. Larger studies are needed to confirm this.

Based on randomized controlled trials (RCTs) with a maximum of 751 patients, the largest of those is meta-analysis found a statistically significant reduction in wound complications with the laparoscopic repair of incisional hernias compared to the open one [20,21].

Several studies have been conducted on the advantages of IPOM plus over open RS approach: in terms of lower risk of surgical-site occurrence, better cosmetic outcome, lower recurrence rate, shorter hospital stay, also faster return to daily activities [22,23]. Therefore, our current analysis confirms the findings of the meta-analysis and studies mentioned previously.

In summary, we are in concordance with International Endohernia Society (IEHS) to recommend IPOM plus for laparoscopic ventral hernia repairs [24].

## Conclusions

1. IPOM plus approach for the ventral and incisional hernias repair is a viable and relatively more safe operation by comparing with open RS repair.
2. IPOM plus approach takes time significantly in 1.3 times less compared to open RS technique.
